# Geographic Trends in Opioid Overdoses in the US From 1999 to 2020

**DOI:** 10.1001/jamanetworkopen.2022.23631

**Published:** 2022-07-28

**Authors:** Lori Ann Post, Alexander Lundberg, Charles B. Moss, Cynthia A. Brandt, Irene Quan, Ling Han, Maryann Mason

**Affiliations:** 1 Department of Emergency Medicine, Feinberg School of Medicine, Northwestern University, Chicago, Illinois; 2 Feinberg School of Medicine, Northwestern University, Chicago, Illinois; 3 Buehler Center for Health Policy and Economics, Chicago, Illinois; 4 Department of Agricultural Economics, University of Florida, Gainesville; 5 Center for Health Informatics, Yale School of Medicine, New Haven, Connecticut

## Abstract

This cross-sectional study examines changes in rates of opioid-involved overdose deaths from 1999 to 2020 in US counties categorized from most urban to most rural.

## Introduction

The US opioid crisis has evolved over time. Ciccarone^1^ posited a theory of 3 overlapping waves of opioid-involved overdose deaths (OODs) based on supply (iatrogenic and new illicit sources) and demand (social, cultural, and technological). Wave 1, in approximately 2000, was prompted by doctors overprescribing opioid painkillers, which was associated with mass addiction.^1^ Wave 2 involved heroin; OODs from heroin escalated in 2007 and surpassed those from prescription opioids by 2015.^1^ Wave 3 involved illicit synthetic opioids, such as fentanyl, the use of which escalated after 2013.^1^ Further evidence suggests a fourth wave, complicated by the addition of stimulants and the COVID-19 pandemic.^2^ To inform prevention and mitigation strategies, this cross-sectional study examined trends in OOD rates in urban and rural US counties during the 4 waves.

## Methods

**Figure 1.**
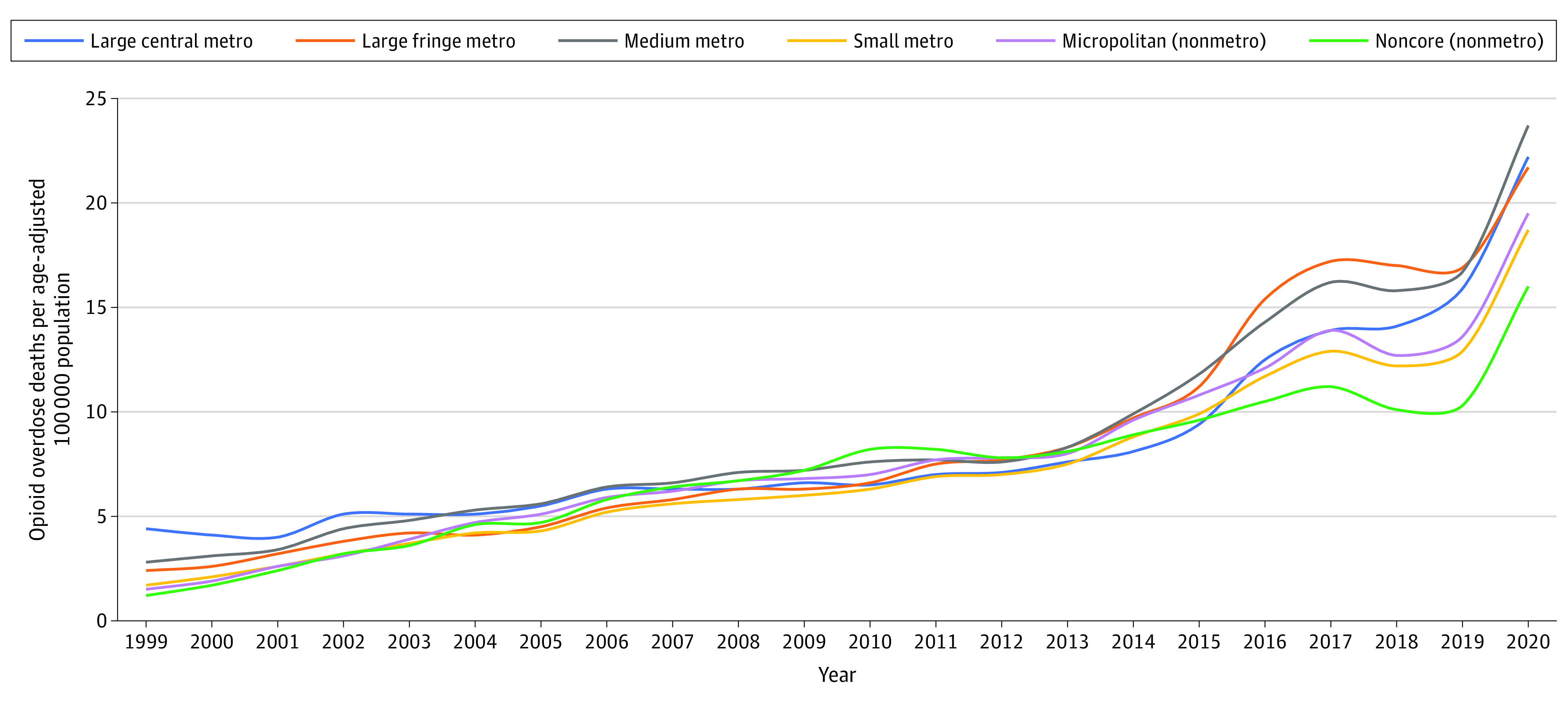
Opioid Overdose Deaths by Urbanicity From 1999 to 2020 Numbers of opioid overdose deaths were generated from the Centers for Disease Control and Prevention’s WONDER Multiple Cause of Death file and were classified using *ICD-10* codes for primary underlying cause of death (X40-44, X60-64, X85, and Y10-14) and for the following: T40.0 (opium), T40.1 (heroin), T40.2 (other opioids), T40.3 (methadone), T40.4 (other synthetic narcotics), or T40.6 (other and unspecified narcotics). Large central metro indicates counties in metropolitan statistical areas (MSAs) with a population ≥1 000 000 that contain all or part of a principal city of the area; large fringe metro, the remaining counties (similar to suburbs) of MSAs with ≥1 000 000 population; medium metro, counties in MSAs with populations of 250 000 to 999 999; small metro, counties in MSAs with populations <250 000; micropolitan, nonmetropolitan counties belonging to a micropolitan statistical area; and noncore, the remaining nonmetropolitan counties. Large central metro is the most urban category and noncore the most rural.

Data included OODs from January 1, 1999, to December 31, 2020, recorded in the Centers for Disease Control and Prevention’s WONDER database for 3147 counties and county equivalents categorized on a 6-point urbanicity scale (most urban to most rural) (Figure 1). OODs were defined using *ICD-10* codes for underlying and multiple causes of death (Figure 1). We followed the STROBE reporting guideline. The institutional review board of Northwestern University exempted the study from approval and waived informed consent because publicly available data were used. We calculated OOD rates as OOD count within a given year and county type, divided by midyear population, multiplied by 100 000. Acceleration (relative change in OOD rate year over year) is expressed as a percentage. Data were analyzed with Microsoft Excel, version 16.61.

## Results

Counties of every urbanicity type experienced statistically significant heterogeneous annual OOD rate growth (Figure 1). Differences in OOD rates by urbanicity were largest at the start and end of the study period. The initial rank order, with urban counties having the highest rates and rural the lowest, reemerged by 2020.

**Figure 2.**
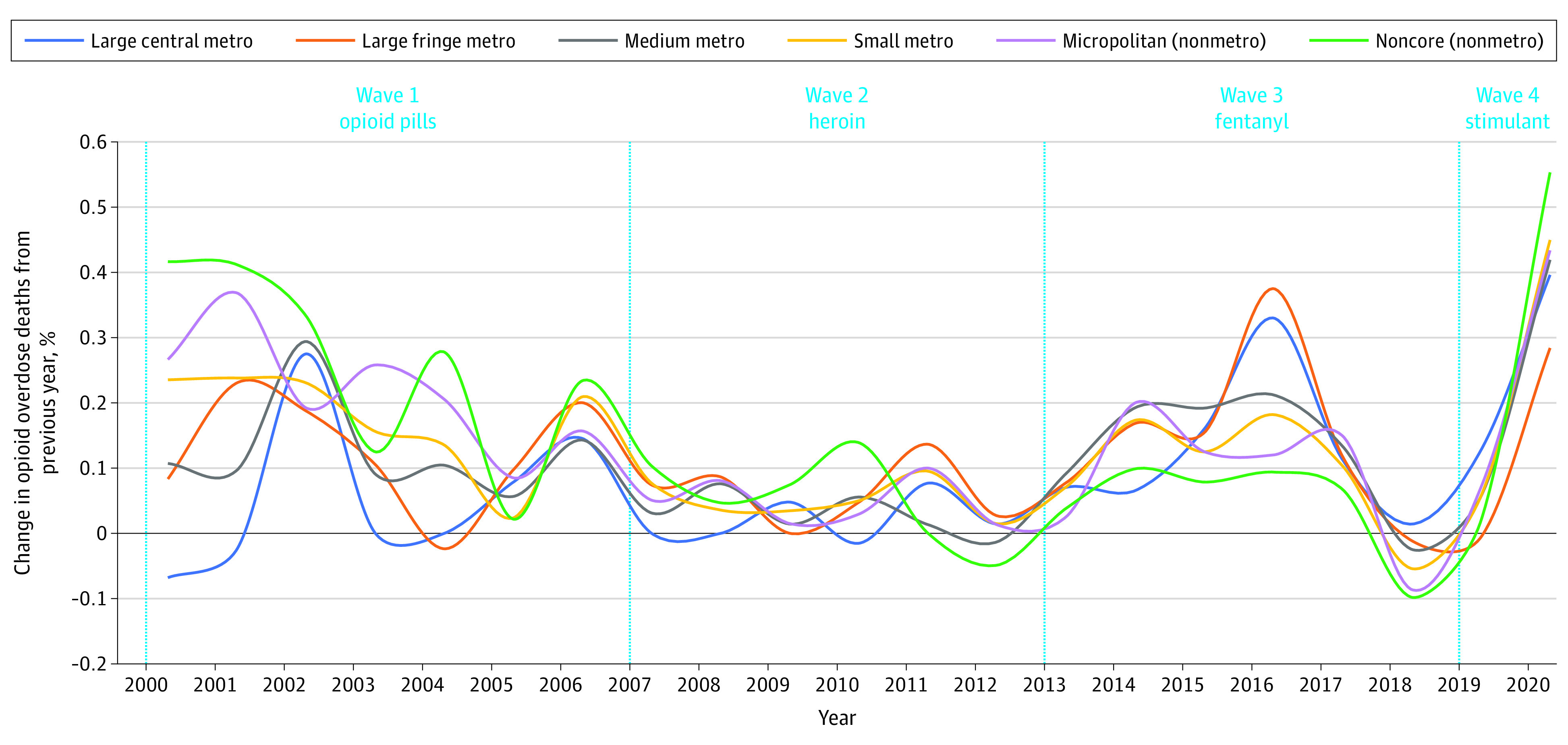
Acceleration Rates of Opioid Overdose Deaths by Urbanicity Large central metro indicates counties in metropolitan statistical areas (MSAs) with a population ≥1 000 000 that contain all or part of a principal city of the area; large fringe metro, the remaining counties (similar to suburbs) of MSAs with ≥1 000 000 population; medium metro, counties in MSAs with populations of 250 000 to 999 999; small metro, counties in MSAs with populations <250 000; micropolitan, nonmetropolitan counties belonging to a micropolitan statistical area; and noncore, the remaining nonmetropolitan counties. Large central metro is the most urban category and noncore the most rural.

In waves 1 and 4, OOD rates were higher in the most urban counties but acceleration rates were higher in the most rural counties (Figure 2). Wave 2 was characterized by approximately linear growth in OOD rates, with diverse trends across urbanicity types. In wave 3, linear growth shifted to nonlinear growth, with 4 years of substantial acceleration across all urbanicity types; OODs from fentanyl increased by a factor of 12. In wave 4, there was marked growth across all urbanicity types.

## Discussion

Overall, OOD rates increased steadily in counties of every urbanicity type, although there were distinct temporal wave patterns by urbanicity. Before 2010, OOD rates accelerated more quickly in rural counties than in urban counties; before 2000, OODs were rare in rural communities, which lacked resources to treat opioid use disorders associated with prescription opioids in wave 1.^3^

Restrictions on synthetic and semisynthetic opioids are associated with increased heroin use, which contributed to wave 2.^4^ OOD rates accelerated more quickly in urban counties during wave 2, beginning approximately in 2013.

From 2013 to 2019, OODs from fentanyl increased 12-fold. This third wave substantially impacted urban and rural counties. The COVID-19 pandemic coincided with a fourth wave marked by worsening of the opioid crisis in all county types.

These results are consistent with the wave theory of Ciccarone.^1,2^ The varied timing of acceleration by urbanicity suggests that policy makers should consider resources and socioeconomic and treatment needs of rural and urban communities as the opioid crisis evolves,^5^ particularly because urban outreach and treatment approaches may not work in rural areas.^3,6^

A limitation is that the data may undercount OODs because death investigation systems vary by state. Not all individuals who die of opioid-involved overdose have opioid use disorder, and deaths in wave 4 are often a function of multiple drug interactions or inclusion of stimulants with synthetic opioids in the nonregulated nonopioid drug supply.^2^ Some OODs are the result of drug interactions or recreational use that may require nonrehabilitative interventions.
